# Optimizing primary healthcare experience: assessing client satisfaction in Kaduna State, Northwest Nigeria

**DOI:** 10.1186/s12875-024-02481-7

**Published:** 2024-06-26

**Authors:** Muhammad Bashar Jibril, Mohammed Nasir Sambo, Hadiza Sulaiman, Hassan Shuaibu Musa, Abubakar Musa, Zaharaddeen Babandi Shuaibu, Lawal Aminu, Yusuf Hassan Wada, Aliko Ahmed

**Affiliations:** 1 National Health Insurance Authority, Abuja, Nigeria; 2 Abubakar Tafawa University, Bauchi, Nigeria; 3 Ahmadu Bello University, Zaria, Nigeria; 4 Society for Family Health, Abuja, Nigeria; 5 University of Cambridge, London, UK

**Keywords:** Clients, Satisfaction, PHC Services, Kaduna, North-west Nigeria

## Abstract

**Background:**

Client satisfaction is a multidimensional construct focusing on clients’ perceptions and evaluations of the treatment and care received. It is one of the factors affecting the outcomes of healthcare and the use of health services. Therefore, we aimed to assess clients’ satisfaction with PHC services in Kaduna State, Northwest Nigeria.

**Methodology:**

A cross–sectional descriptive study was conducted in Kaduna State, Northwest, Nigeria which evaluate the satisfaction of clients and caregivers accessing healthcare in PHC centres. A sample size of 217 was determined using Fisher’s formula, with a multi-stage sampling technique used to randomly select eligible respondents, who have accessed at least a PHC service in any of the PHCs in the State were included in the study, A semi-structured, interviewer-administered questionnaire was administered, and the data collected was analyzed using SPSS version 23.0. Appropriate statistical tests were used to examine the association between dependent and independent variables, while predictor variables that showed significant association with the outcome variables were further subjected to logistic regression analysis, to determine factors that affect clients’ satisfaction with PHC services. Statistical significance was determined at an alpha level set at 0.05 at a 95% confidence interval.

**Results:**

Thirty-one percent of the respondents were satisfied with PHC services in Kaduna State with a mean composite satisfaction score of 3.78 ± 0.67. Age, ethnicity, level of education, and occupational status were factors affecting clients’ satisfaction with PHC services among the respondents. On multivariate analysis, age, ethnicity, educational status, and occupational status were significant factors affecting clients' satisfaction with PHC services. Clients of Hausa/Fulani extraction are one and a half times less likely to be satisfied with PHC services when compared to clients from other tribes [aOR = 1.5, 95% CI (1.21–4.67); *p* = 0.003]. In terms of educational status, clients with formal education are one and a one-third times more likely to be satisfied [aOR = 1.3, 95% CI (0.17–0.94)] with PHC service when compared with their counterparts with informal education (*p* = 0.034).

**Conclusion:**

Clients’ satisfaction with PHC services in Kaduna State, Northwest Nigeria was sub-optimal. Healthcare providers were recommended to improve their attitude bearing in mind clients’ peculiarities.

## Background

Primary Healthcare (PHC) services consist of healthcare services and activities that interface between the community and the healthcare system [1]. It includes all the basic healthcare services to be provided to the community, including immunization, integrated management of childhood diseases, skill birth attendance, maternal care, prevention of mother-to-child transmission, amongst others. It is essential for attaining an acceptable level of health for the public and is also an integral and critical component of the healthcare system of any country. Countries with strong PHC services have healthier populations [2]. This is because the effective provision of efficient PHC services ensures the promotion, protection, and restoration of the health of the community [1]. However, PHC facilities should be accessible to all irrespective of their socio-economic status. Healthcare service utilization is an important determinant of health [3]. The decision to use available health services partly depends on clients’ satisfaction and their perception of the service providers. PHC service utilization has remained limited across the globe, especially in many parts of developing countries [4]. This presents a daunting challenge to Universal Health Coverage (UHC) and in return the attainment of the Sustainable Development Goals (SDG). Studies have attributed many factors to the poor PHC service utilization in sub-Saharan Africa generally, and in Nigeria specifically [5]. These factors include financial means to pay for such services; means to reach and use the services; confidence in the quality of the care provided and ability to receive care without compromising privacy; confidence in the ability to communicate with healthcare service providers, and overall clients’ satisfaction with services provided in PHC facilities [1]. Client satisfaction is defined as judgments made by the recipients of healthcare as to whether their expectations have been met [6]. It is a multidimensional construct that focuses on clients’ perceptions and evaluation of the treatment and care received [7, 8]. A relationship exists between clients’ satisfaction and healthcare utilization, and clients’ satisfaction determines future access to health services. Clients’ satisfaction has long been considered an important component when measuring health outcomes and quality of care [6]. It is one of the factors affecting the outcomes of healthcare and the use of health services. Hence, the level of clients’ satisfaction is one of the mechanisms used in measuring the level of quality of healthcare service delivery [9] and addressing clients’ expectations was found to be associated with high client satisfaction and better health outcomes [10]. Clients are by far the best judges since they accurately assess and provide inputs which can help in the overall improvement of quality healthcare provision through the rectification of the system weaknesses by the concerned authorities [11]. However, clients' perceptions about healthcare service delivery as well as health systems have been largely ignored by government and healthcare managers in developing countries [12], and healthcare providers lack the awareness and adequate training to address clients’ expectations [6]. To improve the quality of provision and outcome of care, predictors of dissatisfaction must be identified and eliminated.

A report by the WHO African region puts the level of PHC utilization on the continent at 5–7% [13]. This translates to about 93–95% underutilization of the services despite the monumental budgetary expenditure on PHCs.Clients frequently bypass PHC facilities in favour of higher-level hospitals despite the substantial additional time and financial costs. This results in over-burdened and overstretched facilities with a consequent reduction in the quality of services, and of course clients’ dissatisfaction with healthcare service delivery at higher levels of care [14]. In Kenya, Namibia and Sri Lanka, more than 50% of the population bypasses PHC facilities for antenatal care (ANC), immunization services, and treatment of childhood illnesses [15]. In Nigeria where PHC is the bedrock of its health system; and almost half a decade after the Alma-Ata declaration, the health indices are still poor, with a large body of evidence suggesting widespread under-utilization of PHC services. The extent of poor utilization of PHC services in the nation generally, and the northern states in particular was appalling [5]. In Nigeria, particularly in Kaduna State where the present study was carried out, studies have shown on the level of utilization of PHC services and the factors that have affected the utilization [16], with majorly dissatisfied with the services being offered. This is due to the non-availability of trained personnel, attitudes of staffs, waiting time and non-availbility of diagnostic tools. Studies conducted in other part of Nigeria, relating to clients’ satisfaction revealed that overall client satisfaction ranges between 62.6%—90% [17, 18] with some of the clients being dissatisfied with PHC services. However, the respondents showed an average level of dissatisfaction with the general cleanliness of the facility and a relatively higher level (64%) of dissatisfaction with waiting areas [19, 20]. Studies also revealed that respondents were dissatisfied with distance of the facility with their dwellings, working hours of facilities, language barriers, attitude of healthcare workers, client–provider interpersonal interaction and relationship, hospital facilities, missing records, quality access to care, and cost of treatment [21–23]. Clients often make more payments for repeated or replacement of the investigations or procedures without convincing explanation or apology from the healthcare providers. This has over time caused untold hardship, disappointment, and often embarrassment to the clients and, or their relatives, with resultant postponement, suspension, or outright termination of medical care. Treatments are often not fully explained to the clients or at best without courtesy and human dignity [24]. This has resulted in deaths, disabilities, complications, treatment failures, or loss of financial resources that could have been prevented only if there had been good communication between healthcare workers and clients [22]. Additionally, lack of confidentiality and poor interpersonal communication between clients and the healthcare providers have worsened the problems of clients with the resultant absence of, decreased, or lower levels of reported satisfaction among the majority of such clients [25]. These realities have resulted in poor utilization of PHC facilities and services by the intended or prospective client. The conduct of this study is therefore justified againts the background, because there exists a relationship between clients’ satisfaction and healthcare utilization. In the same vein, clients’ satisfaction determines future access to health services [26]. Similarly, understanding the determinants of clients’ satisfaction helps healthcare systems to address clients’ needs and adjust their expectations to realistic and achievable targets or expectations in line with the vision outlined in the current Nigerian National Health Act [27] and National Policy Framework [28]. Furthermore, many of the well-known frameworks to structure health system thinking, such as WHO’s building block and the “control knobs” framework include a measure of clients’ subjective evaluation of health services, such as satisfaction or responsiveness as major components of the main health systems outcomes [29]. Furthermore, in developed countries and a few developing ones, clients’ satisfaction is a widely recognized factor used to assess the quality of PHC service delivery [26]. This, however, has not been fully studied in our climes [30]. The low clients’ satisfaction with PHC services is to a certain extent associated with poor PHC service utilization in Nigeria generally and in Kaduna state specifically. However, the association of poor clients’ satisfaction with PHC services has not been documented in this environment. It is therefore hoped that the findings from this study will not only stimulate further research but will also form the basis for a series of interventions to bridge the gap in the existing body of knowledge concerning clients’ satisfaction. The information to be obtained through assessing clients’ satisfaction will determine the level of care to be delivered by healthcare providers and as a result, would be used to hold health providers accountable for their services. The information generated from this study will not only be used as a tool for decision-making but will also be used as a reference point to make further recommendations on improving the experience of clients being seen at PHC facilities in Kaduna state specifically, and Nigeria generally. This study, therefore, aims to assess clients’ satisfaction with PHC services in Kaduna State, Northwest Nigeria.

## Methodology

### Study design and area

The study was a cross-sectional descriptive study conducted in Kaduna State, Northwest Nigeria. Kaduna State is located in the geographic ‘heart’ of Nigeria where it shares borders with five other States—Kano, Katsina, Niger, Plateau, Nasarawa, in addition to Nigeria’s federal Capital Territory – Abuja. Kaduna occupies an area of approximately 48,473.2 square kilometers and has a projected 2023 population of about 10 million (projected from the 2006 census), with an annual growth rate of 2.5%. The state is mostly populated by Hausa, Fulani, Gwari, Katab, Bajju, Kataf, Kagoro, Moro’a Jaba, Gbagyi, Kanninkon, Ninzam, Chawai, Atyap, and Ham Kurama ethnic communities, with up to 36 other indigenous ethnic groups found in different parts of the State. The state also has a considerable population of other Nigerians who settled in when its capital city of Kaduna served as the capital of the defunct Northern Region. Hausa and English are the most common languages of communication. Politically, the state is divided into three (3) senatorial zones—Northern, Central and Southern zones. There are 23 Local Government Areas (LGAs) in the state with 255 political wards and the healthcare delivery system in Kaduna State is broad-based, comprising public, private-for-profit, and private-not-for-profit mainly faith-based health facilities. The Federal Ministry of Health provides Tertiary Healthcare services in the State through the five (5) federal health facilities in the State comprising Ahmadu Bello University Teaching Hospital (ABUTH), National Eye Care Center, National Ear Care Center, Federal Neuropsychiatric Hospital, and the 44 Military Reference Hospital. The Kaduna State Ministry of Health, on the other hand, provides secondary healthcare services through the 30 General and Specialist Hospitals, while the Local Governments provide primary healthcare through the 1,068 functional PHC facilities in the State. In addition, there are 656 private health facilities, and over 2,500 registered patent medicine vendors and Traditional Birth Attendants (TBAs) within the State [31]. The State has eight (8) academic institutions and four (4) post-basic training programs for human resources training and development.

### Sampling and data collection

The minimum sample size for the study was estimated using Fisher’s formula with a Proportion (p) of 0.83 from a previous study [18], a reliability coefficient of 1.96 at 95% Confident Interval, and a degree of freedom of 0.05. We use Fisher’s formula for this study because it provides us with the best option to do a simple random sampling in order to estimate the value of the population’s satisfaction to PHC services. Hence an estimated minimum sample size of 217 was obtained for the study. The study population comprised clients and caregivers accessing healthcare in PHC centres owned by the Kaduna State Primary Health Care Development Board (PHCDB). Clients and caregivers accessing care in public PHC facilities in Kaduna State who have accessed at least a PHC service in any of the PHC centres within the State in the last year were included in the study, while those with mental health problems, emergency conditions, and those with hearing or speech difficulty that may limit or hinder their ability to hear or respond to the data collection process were excluded from the study. A multi-stage sampling technique comprising five stages (selection of political ward, selection of LGAand selection of clients to respond to exit questionnaire) was used for the study. The number of clients to whom the clients' exit questionnaire was administered was proportionately determined based on the average clients’ turnover per PHC centres. A pre-tested, semi-structured, interviewer-administered questionnaire adapted from the Long-Form Patient Satisfaction Questionnaire (PSQ-III) [32], Short-Form Patient Satisfaction Questionnaire (PSQ-18) [33], and Patient Satisfaction Questionnaire (PSQ) [34] and modified based on the specific objectives of the study was electronically administered with handheld Android devices using Open Data Kit (ODK) software to the randomly sampled respondents by a team of ten (10) research assistants comprising of two (2) resident doctors and eight (8) Community Health Officers (CHOs) from the Department of Community Medicine, Ahmadu Bello University Teaching Hospital Zaria. The psychometric properties of the questionnaire were evaluated with face and content validity, with the face validity assessed by five staff of the Department of Community Health, Ahmadu Bello Zaria (2), Ministry of Health (1) and National Primary Health Care Development Agency (2). They commented on the relevance, reading level and ambiguity using a five-point Linkert scale. The content validity was assessed by sharing the draft questionnaire with four professors of community health to provide feedback on the level of clarity, relevance, and importance using a Linkert scale of 1–5. It was then tested quantitatively using Scale-content validity index (only item with < 0.90 were retained) and Item-content validity index (only items with value < 0.79 were retained). The reliability of the questionnaire was evaluated using internal consistency (Cronbach’s alpha coefficient with a value of 0.7 were considered good) and stability methods (intraclass correlation coefficient of 0.7 was considered good). The research assistants were the recruited based on their previous experience in data collection in studies similar to this study and trained on the objectives of the study, questionnaire administration, ethical issues in research, ODK, clients’ satisfaction, and the study protocol before the data collection. The researchers supervised the data collection process and ensured that the research protocol was strictly adhered to. Daily review meeting of research assistants and researchers was held during the data collection to ensure consistency and completeness of questionnaires as well as identify and address challenges encountered during the data collection to re-strategize for the subsequent days.

### Data management and analysis

All electronic questionnaires retrieved were assessed and found to be correctly completed. Data analysis was done under the supervision of an experienced statistician, using the computer software, IBM Statistical Package for Social Sciences (SPSS) version 23.0 into which the data was exported. Data analysis was started by computing the frequencies and percentages of respondents’ socio-demographic characteristics. Clients’ satisfaction with PHC was assessed using a set of 27 questions across four (4) major domains based on a 5-point Likert Scale. An average combined composite score of 1 to 5 was expected for each question based on the responses obtained. Using the average respondents combined composite score, clients’ satisfaction with PHC services was assessed. Respondents with an average combined composite score of ≥ 3 were considered to be satisfied with PHC services, while those with a combined composite score of < 3 were considered to be dissatisfied with PHC services provided. For univariate analysis, descriptive statistics was conducted using mean and standard deviation (for normally distributed continuous data). Simple frequencies and percentages were reported for categorical data. Data was presented in the form of tables and charts using Microsoft Office Excel 2016. Bivariate analysis was used to examine the association between dependent and independent variables. A chi-square test of association was done to determine the significant (*p* ≤ 0.05) associations between independent and dependent variables. Predictor variables that showed significant association with the outcome variables were further subjected to logistic regression analysis, to determine factors that affect clients’ satisfaction with PHC services. Statistical significance was determined at an alpha level set at 0.05 at a 95% confidence interval. The findings were summarized using appropriate tables and relationships between variables were determined using appropriate test statistics. Results were considered statistically significant if the *p*-value was ≤ 0.05.

## Results

A total of 217 questionnaires were administered, out of which 198 were retrieved. This gives response rates of 91.24% for the study.

### Clients’ socio-demographic factors

Table 1 shows that the sociodemographic data of the respondents with a predominantly young population, majorly aged between 30–39 (43.4%)with their mean age ± Standard Deviation, SD as 40.07 ± 7.69 years. Most 172 (86.8%) of the clients were females and are majorly Hausa by tribes (143 (72.4%). Similarly, Muslims were predominant among the respondents (176 (88.7%). Also, most respondents (166 (84.0%) were ever married. In terms of educational status, 78 (39.5%) of the respondents had secondary school as their highest level of education. A high proportion of the clients (52 (26.4%) were petty traders. However, 146 (73.7%) of the clients had an average monthly income below ₦50,000.00 with a median of ₦10,000.00 (interquartile range ₦16,000.00).

**Table 1:** Socio-demographic characteristics of respondents

Variable	Frequency (%) (*n* = 198)
**Age Group**	
20 – 29	11 (5.7)
30 – 39	86 (43.4)
40 – 49	82 (41.5)
50 – 59	17 (8.5)
60 – 69	2 (0.9)
**Mean ± SD**	**40.07 ± 7.69**
**Sex**	
Male	26 (13.2)
Female	172 (86.8)
**Ethnicity**	
Hausa	143 (72.4)
Fulani	20 (10.3)
Yoruba	15 (7.4)
Igbo	13 (6.4)
Others	7 (3.6)
**Religion**	
Islam	176 (88.7)
Christian	22 (11.3)
**Occupation**	
Not Employed	49 (24.5)
Petty Trading	52 (26.4)
Civil Servants	37 (18.9)
Teaching	17 (8.5)
Tailor	13 (6.6)
Others	30 (15.1)
**Marital Status**	
Never Married	32 (16.0)
Ever Married	166 (84.0)
**Educational Level**	
None	5 (2.6)
Informal	9 (4.6)
Primary	19 (9.6)
Secondary	78 (39.5)
Tertiary	79 (40.1)
Others	7 (3.6)
**Average Monthly Income**	
≤ ₦50,000.00 (≤ 70)	146 (73.7)
₦500,001.00 – ₦100,000.00 (70 – 139)	32 (16.0)
₦100,001.00 – ₦150,000.00 (140 – 210)	11 (5.7)
> ₦150,000 (> 210)	9 (4.7)
**Median (IQR)**	10,000 (16,000)

### Clients’ satisfaction with PHC services

Table 2 showed that clients had a satisfaction score of 4.13 ± 0.95 with the extent to which they felt that they could have refused any proposed test, treatment, or procedure if they wanted to.

**Table 2:** Clients’ composite satisfaction score with PHC services

Clients’ satisfaction with PHC services	Mean ± SD (*n* = 198)
**Clients’ satisfaction with PHC facility processes**	
Clients’ satisfaction with the responsiveness of health workers to their needs in the facility: I am satisfied with the responsiveness of the health workers in this facility to my needs?	3.66 ± 0.73
Clients’ satisfaction with health workers' explanation of information about their treatment: I am satisfied with how information about my treatment was explained to me by the health workers?	3.69 ± 0.68
Clients’ satisfaction with health workers' general helpfulness in the facility: I am satisfied with the helpfulness of the health workers generally in the facility?	3.74 ± 0.57
Clients’ satisfaction with health workers' respect for their privacy in the facility: I am satisfied with the respect to my privacy by the health workers?	3.75 ± 0.66
Clients’ satisfaction with health workers respect to their cultural or religious needs in the facility: I am satisfied with how well my cultural or religious needs were respected by the health workers?	3.66 ± 0.58
Clients’ satisfaction with their general personal safety in the facility: I am satisfied generally with my personal safety in the facility?	3.75 ± 0.68
Clients’ satisfaction with their being treated with respect by the health workers in the facility: I am satisfied with my being treated with respect by the health workers in the facility?	3.81 ± 0.54
Clients’ satisfaction with their being given the opportunity by health workers in the facility to ask questions about their condition or treatment: I am satisfied with the opportunity given to me to ask questions about my condition or treatment?	3.81 ± 0.66
Clients’ satisfaction with their being involved by health workers in the facility in decisions about their care: I am satisfied with the way the health workers involved me in decision about my care?	3.86 ± 0.77
Clients’ satisfaction with health workers’ willingness to listen to their health care problems: I am satisfied with the willingness of the health workers to listen to my health care problems?	3.78 ± .063
Clients’ satisfaction with health workers' response to their health problems: I am satisfied with how well health workers responded to my health problem?	3.80 ± 0.67
Clients’ satisfaction with the extent to which health workers checked that they understood the information given to them: I am satisfied with the extent to which the health workers checked that I understood the information given to me?	3.75 ± 0.63
Clients’ satisfaction with the extent to which health workers checked that they were taking their medication(s) or not: I am satisfied with the extent to which the health workers checked that I am taking my medication(s) or not?	3.92 ± 0.69
Clients’ satisfaction with the extent to which health workers take a history of their dietary needs: I am satisfied with the extent to which the health workers take history with regards to my dietary needs?	3.95 ± 0.84
Client’s satisfaction with the extent to which health workers asked them if someone else such as a family member could be given information about their condition: I am satisfied with the extent to which the health workers asked me if someone else such as a family member could be given information about my condition?	4.04 ± 0.92
Clients’ satisfaction with the extent to which health workers asked them if they understood the information given to them: I am satisfied with the extent to which the health workers asked me if I understood the information given to me?	3.88 ± 0.67
Clients’ satisfaction with the extent to which they felt that they could have refused any proposed test, treatment, or procedure if they wanted to: I am satisfied with the extent to which I felt that I could have refused any proposed test, treatment or procedure if I wanted to?	4.13 ± 0.95
Clients’ satisfaction with the extent to which they felt that you could have asked for a second opinion about their proposed test, treatment, or procedure if they wanted to: I am satisfied with the extent to which I felt that i could have asked for a second opinion about my proposed test, treatment or procedure if I wanted to?	4.11 ± 0.83
Clients’ satisfaction with health workers’ effort to discuss the benefits and risks of their treatment: I am satisfied with the effort made by health workers to discuss the benefits and risks of my treatment?	4.04 ± 0.76
**Clients’ satisfaction with PHC care and treatment management**	
Clients’ satisfaction with health workers’ respect for their right to have an opinion: I am satisfied with regards health workers respect for my right to have an opinion?	3.86 ± 0.71
Clients’ satisfaction with the respect they were shown while being interviewed or examined by health workers: I am satisfied with the respect I was shown while being interviewed or examined by health workers?	3.83 ± 0.58
Clients’ satisfaction with the consideration and politeness they were shown by health workers: I am satisfied with the consideration and politeness I was shown by health workers in the facility?	3.75 ± 0.65
Clients’ satisfaction with the tone of voice used by health workers when interviewing or examining them so that others couldn’t overhear their discussion: I am satisfied with the tone of voice used by the health workers when interviewing or examining me so that others couldn’t overhear my discussion?	3.81 ± 0.71
**Clients’ satisfaction with the physical aspect of the PHC facility**	
Clients’ satisfaction with the help they got from signposts while getting around the facility: I am satisfied with the help I got from the signposts while getting around the hospital?	3.97 ± 0.88
Clients’ satisfaction with the cleanliness of the hospital surroundings: I am satisfied with the cleanliness of the hospital surrounding?	3.87 ± 0.69
Clients’ satisfaction with the cleanliness of the consultation rooms in the facility: I am satisfied with the cleanliness of consultation rooms in the hospital?	3.75 ± 0.62
**Clients’ general satisfaction with PHC service delivery**	
Clients’ satisfaction generally with the health care services they received in this facility: I am satisfied generally with the health care services I received in this facility?	3.60 ± 0.56
**Mean satisfaction score**	**3.78 ± 0.67**

Satisfaction score based on a 5-point Likert Scale

### Clients’ Satisfaction with PHC Services

**Fig. 1:**
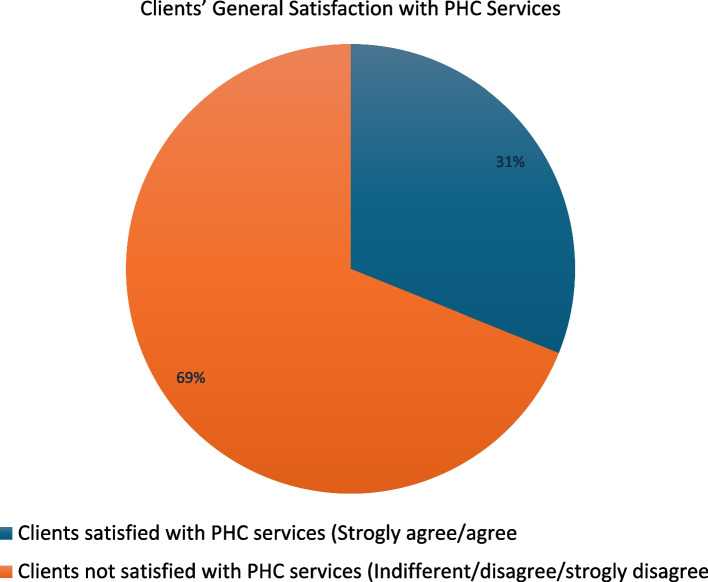
Clients general satisfaction with PHC services

Figure 1 revealed the client’s satisfaction with PHC services. It shows that only 31.1% of the clients were satisfied with PHC services who visited a facility for their healthcare needs.

### Factors affecting clients’ satisfaction with PHC services

At bivariate level, Table 3 showed that clients’ satisfaction with PHC services was significantly affected by age (0.026), ethnicity (0.003), religion (0.030), occupation (0.002), highest level of education (0.020), and average monthly income (0.001).

**Table 3:** Socio-demographic factors affecting clients’ satisfaction with PHC services

	**Clients Satisfied with PHC Services**	**Clients Not satisfied with PHC Services**		
**Variable**	**Mean ± SD**	**Mean ± SD**	**F(df)**	*P-value*
**Age Group**				
< 20	3.83 ± 0.408	4.00 ± 0.0	3.152(3)	**0.026**
20 – 29	3.59 ± 0.50	3.52 ± 0.54		
30 – 39	3.55 ± 0.63	3.58 ± 0.64		
40 – 49	3.90 ± 0.88	3.88 ± 0.35		
**Sex**				
Male	3.43 ± 0.51	3.55 ± 0.52	-1.121	0.264
Female	3.64 ± 0.60	3.60 ± 0.57		
**Ethnicity**				
Hausa	3.63 ± 0.52	3.58 ± 0.54		
Fulani	3.36 ± 0.51	3.45 ± 0.52		
Yoruba	3.33 ± 0.49	3.50 ± 0.58	4.083(4)	**0.003**
Igbo	4.00 ± 0.76	4.20 ± 0.84		
Others	4.00 ± 1.23	3.67 ± 0.58		
**Religion**				
Islam	3.56 ± 0.52	3.56 ± 0.54	-2.361	**0.030**
Christian	4.00 ± 0.95	4.17 ± 0.75		
**Occupation**				
None	3.70 ± 0.48	3.73 ± 0.47		
Petty trading	3.54 ± 0.64	3.55 ± 0.67	4.041(5)	**0.002**
Teaching	3.44 ± 0.53	3.29 ± 0.49		
Civil Service	3.50 ± 0.51	3.43 ± 0.51		
Housewife	3.44 ± 0.63	3.50 ± 0.65		
Others	4.00 ± 0.56	4.00 ± 0.40		
**Marital Status**				
Single	3.67 ± 0.58	3.67 ± 0.58	0.269	0.788
Married	3.61 ± 0.60	3.59 ± 0.57		
**Highest Level of Education**				
Informal	3.67 ± 0.52	3.86 ± 0.378		
Primary	3.80 ± 0.42	3.64 ± 0.51	2.999(4)	**0.020**
Secondary	3.43 ± 0.50	3.46 ± 0.54		
Tertiary	3.69 ± 0.68	3.69 ± 0.62		
Others	3.75 ± 0.50	3.75 ± 0.50		
**Average monthly Income**				
≤ 100,000	3.63 ± 0.57	3.75 ± 0.55	3.254	**0.001**
> 100,000	3.47 ± 0.52	3.20 ± 0.41		

### Multivariate (Logistic Regression Analysis) of socio-demographic factors that affect clients’ satisfaction with PHC services

On multivariate analysis, Table 4 showed that age, ethnicity, educational status, and occupational status were significant factors affecting clients' satisfaction with PHC services. Table 4 showed that clients of 30 years and above had almost a three-fold likelihood of being satisfied with PHC services compared to those below 30 years of age. Clients of Hausa/Fulani extraction are one and a half times less likely to be satisfied with PHC services when compared to clients from other tribes. In the same vein, clients not gainfully employed had twice as much likelihood of being satisfied with PHC services as compared to their gainfully employed counterparts. In terms of educational status, clients with formal education are one and a one-third times more likely to be satisfied [aOR = 1.3, 95% CI (0.17–0.94)] with PHC service when compared with their counterparts with informal education (*p* = 0.034).

**Table 4:** Determinants of clients’ satisfaction with PHC services

Variables	Crude OR (95% CI)	Adjusted OR (95% CI)	*p*-value
**Age**			
< 30 years	1		
≥ 30years	1.4 (0.98 – 2.06)	2.9 (1.53 – 3.33)	0.036*
**Ethnicity**			
Hausa/Fulani	1		
Others	1.2 (0.89 – 2.48)	1.5 (1.21 – 4.67)	0.002*
**Religion**			
Islam	1		
Christianity	1.4 (0.67 – 3.92)	2.1 (0.17 – 1.94)	0.410
**Highest level of Education**			
Informal	1		
Formal	1.6 (0.98 – 2.65)	1.3 (1.14 – 5.37)	0.034*
**Occupation**			
Employed	1		
Unemployed	7 (0.41 – 0.97)	1.9 (1.33 – 5.62).	0.023*
**Monthly Income**			
< Minimum wage	1		
> Minimum wage	1.6 (0.37 – 1.59)	0.4 (0.17 – 0.91)	0.69

^*^Statistically significant

## Discussion

Clients’ level of satisfaction with PHC was generally found to be 31.1%. However, this study also reported a higher average mean score above 3.6 in all the four dimensions being assessed. The findings of this study are quite low in comparison with reported studies which reported 78.5%, 83% and 83% of clients with overall satisfaction with services received, respectively [18, 35, 36]. These findings are equally lower than findings from a survey conducted in Thailand with 86.7% of clients satisfied with services [37]. This is also lower than findings reported which ranges from 63–95% in Haryana (79.3%); in Lucknow, (81.6%); in Kashmir (72%); in India (88%) and (95%); and in Andra Pradesh (63%) [38–43]. In contrast to this, however, a study conducted at Indira Gandhi Memorial Hospital, reported lower levels of clients’ satisfaction (10.4%) in comparison with the findings of this study [44]. It is important to note, however, that expressed opinions may not reflect the true feelings of clients about their satisfaction with healthcare services received. Furthermore, confusion may arise even when opinions are expressed honestly; for instance, long waiting times may induce patients to regard health providers as discourteous or lacking in skill thereby grading them low on satisfaction scores. Indeed, waiting time was used in some studies as a means of judging health providers' skills and knowledge. Apart from variations in the way services are delivered, differences in the study population and hence patient expectations could affect satisfaction levels. The latter could also be affected by sociocultural differences and variations in levels of literacy. The cultural milieu and relatively lower level of literacy of our catchment population could have affected their level of satisfaction. In addition, variations in methodology and timing of the study could explain some of the differences. For example, in many studies, age were found to be the strongest predictor of patient satisfaction across most dimensions [43–46]. These variation in satisfaction across the four dimension and the predictors might be associated with differences in healthcare systems, cultural contexts or methodologies, as these calls for caution in comparing our findings with previous studies. In Northern Nigeria where our study was conducted, there is a wide disparity between PHC services, which could have contributed in the high difference in general satisfaction and expectation among patients who participated in our study. These factors might include out-of-pocket cost they might not expect, but satisfactory with the four domains. This is similar to a study which reported patient satisfaction in a tertiary public hospital in Nepal [41] and India [40, 42]. It could probably be concluded that the reason why most satisfaction studies in developing countries have high reports of low levels of satisfaction among clients could be due to inadequate, inappropriate, incompetent, poorly mixed, and poorly remunerated health workforce. Overall satisfaction score and mean score for different domains of clients’ satisfaction were also found to be similar. The study revealed that “Clients’ satisfaction with the extent to which they felt that they could have asked for a second opinion about their proposed test(s), treatment or procedure(s) if they wanted to”, “Clients’ satisfaction with health workers’ respect for their rights to have an opinion” and “Clients’ satisfaction with the cleanliness of the hospital surrounding” had the highest mean satisfaction scores among the constituents of “Clients’ satisfaction with PHC facility processes”, “Clients’ satisfaction with PHC care and treatment management” and “Clients’ satisfaction with the physical aspect of PHC facility” respectively. This finding is like that of a study on patient satisfaction in primary healthcare services in Egypt [45], which indicated that the majority of clients had high satisfaction scores for PHC facility process, care, and treatment management as well as aspect of PHC facility. The study further revealed higher satisfaction scores with components of clients’ satisfaction with PHC facility processes in comparison with components of other constituents of clients’ satisfaction with PHC services. These findings could be explained by the fact that most of the clients find consultations with the healthcare providers fulfilling their desire to come to the facility. This is where they interact one-on-one in private, and establish relationships with the providers, which is vital for the success of primary healthcare. The utmost communication and information sharing with the providers thereby gives them renewed confidence about whatever ailment they came in with. According to a study [46], non-understanding of clients’ information needs will affect any meaningful service improvement made. These characteristics are of immense importance in contributing to clients’ satisfaction with, and ratings of services received. In another study [47] on patient experiences about participants and healthcare service delivery characteristics, it was reported that for any meaningful success to be recorded, primary healthcare providers must establish relationships with their clients [47]. From the study findings, the clients were most pleased with the provision of information. This concurs with the findings of a study on advice to clients in Swedish primary healthcare, which found that provision of information is ranked higher by clients in terms of factors contributing to their satisfaction [48]. Another study also showed that clients value information highly, as satisfaction in this regard correlated strongly with the amount of information clients received from their Health providers [49]. Another finding of this study indicated that the majority of the clients have high satisfaction scores with components of clients’ satisfaction with the PHC facility process associated with the communicative behaviours of PHC health providers. These findings highlight two points. Firstly, the satisfaction rate of clients is not too bad. The reason may be that being satisfied is a subjective variable and many factors could influence clients’ satisfaction. Thus, the high satisfaction score of clients is not necessarily indicative of the optimum or high quality of healthcare. On the other hand, high clients’ satisfaction may be due to the low expectations of clients, or it could even be influenced by some kind of interviewers’ manner. Therefore, it is argued that one cannot assume the higher quality of healthcare services leading to higher satisfaction levels and/or low scores of satisfactions should not be considered as lower quality of health services, especially in climes where the general population is not well-informed about healthcare quality standards. A study has supported these findings [50]. The second point is that the clients’ satisfaction level could be improved through better training on IPCs. This is because client/provider IPCS training could improve knowledge and skills of IPC among PHC providers and enable them to communicate with their clients more effectively. Other studies have shown that IPCS training could improve clients' satisfaction [51]. In similar research, contrary results were obtained, and this study observed on bivariate analysis that age, ethnicity, religion, occupation, highest level of education, and average monthly income significantly affect clients’ satisfaction with PHC services [50]. However, the results of studies examining the influence of socio-demographic characteristics on patients' satisfaction are varied. A meta-analysis has also reported that patients' demographics are a minor factor in their satisfaction, while another two-level analysis [52, 53] and concluded that clients’ socio-demographic profiles represent 90—95% of the variance in rates of satisfaction. The findings of this study are similar to a study [54] that revealed that educational and occupational status appeared to be statistically associated with satisfaction factor score. This is also in agreement with findings that reported from their study that age significantly affect clients’ satisfaction with PHC services, with clients sixty-year-olds or older being more satisfied (75% vs. 58%) than those below the age of 60 years [51]. Findings of this study was in accordance with the results of studies [52, 53] that revealed a statistically significant relationship between patients’ educational level and the overall score of their satisfaction with services. On the contrary, findings of this study vary with those that show that none of the socio-demographic characteristics significantly relate to patients' satisfaction [55]. This might suggest the universality of man's expectations and demands on health facilities which are independent of these socio-demographic factors. Clients 30 years and above were found to be more likely to be satisfied with PHC services than their counterparts who were less than 30 years of age. In the same vein, clients of Hausa/Fulani extraction are one and a half times less likely to be satisfied with PHC services in comparison with clients of other ethnicities. The study further revealed that clients with informal education are one and a one-third times less likely to be satisfied with PHC services compared to those with formal education. Similarly, those unemployed are twice as likely to be satisfied with PHC services than those employed. Similar to the findings of this study; studies conducted have observed that the clients’ satisfaction scores improve with increasing age [55–58]. In this study, it was found that those with informal education were less satisfied with healthcare facilities when compared to those with any formal education. This finding may be due to the fact that the less literates are more suspicious of the healthcare facilities and thus are less satisfied with healthcare services provided by the facilities. Similar to our findings, some studies have also observed that those unemployed tend to be more satisfied, as compared to those employed. However, some of the limitations of this study include recall bias because of the retrospective nature of some of the questions asked, willful misstatements, and reluctance by some respondents to admit dissatisfaction with services received in PHC facilities because of fear of being victimized or denied access at the point of care by healthcare providers. Our study may also not be generalized in the at sub-national levels as the State seems to have differences in availability of human resources, healthcare service quality and diagnostics. However, our study can be applicable towards improving healthcare services in Nigeria as they provide similar types of services. Also, there could be a number of patients satisfaction dimensions we didn’t include in our questionnaire, especially looking at the supply side that may influence service delivery. Even though we didn’t collect any data related to the supply side factors, our study provides a baseline for future study on healthcare workers’ attitude toward their work, incentives and remuneration that eventually affect patient satisfaction. We also couldn’t assure that the correlates of the patient satisfaction in our study are indeed causal, due to the cross-sectional nature of the study. A more robust study which follows the client/patient for a number of visit and have several controlled variables can give a more causal relationship including the supply-side dynamic of the PHC service. In our study, we use client rather than patient—which in some setting is termed as “derogatory language”. We also use client satisfaction as adapted from studies, but the section is more related to the experiences and evaluations of patients. Therefore, we recommend that readers take a more look at the finding of each item instead of those based on the mean score of the four dimensions. Despite limitations relating to this study, association or predictors can be used to initiate some policy dialogue and influence the quality of healthcare delivery at PHC level in Nigeria. It will also be a good resource to formulate plans and programs to improve client satisfaction in PHC levels.

## Conclusion and recommendation

This study has established that about one-third (31%) of the respondents were satisfied with PHC services in Kaduna State, Northwest Nigeria, with a mean average composite satisfaction score of 3.78 ± 0.67. The study further revealed that clients were more satisfied with the physical aspect of PHC facilities than with PHC facility processes; PHC care and treatment management; and general PHC service delivery. The study also found that socio-demographic factors like age, ethnicity, level of education, and occupation affect clients’ satisfaction with PHC services in Kaduna State, Northwest Nigeria. Consequently, it was recommended that there is a need to encourage PHC providers to improve their attitude towards clients and devise holistic (structure, process, and health outcome) approaches to client care bearing in mind political, economic, socio-cultural, as well epidemiological peculiarities of clients. Similarly, the healthcare providers should be motivated to provide services, bearing in mind different demands, attitudes, and perceptions of clients with different socio-demographic profiles. Future research designs that incorporate the supply-side dynamics and address a wide range of our limitation will be important to advance plans and programs to improve client satisfaction at PHC levels.

## Data Availability

Data or supplementary information files are provided upon request from the corresponding author.
